# Quality of life after carotid endarterectomy: a review of the literature

**DOI:** 10.1007/s13760-017-0811-x

**Published:** 2017-06-21

**Authors:** Mariusz Chabowski, Anna Grzebien, Agnieszka Ziomek, Karolina Dorobisz, Michał Leśniak, Dariusz Janczak

**Affiliations:** 1 Division of Surgical Specialties, Department of Clinical Nursing, Faculty of Health Science, Wroclaw Medical University, 5 Bartla Street, 51-618 Wroclaw, Poland; 2 Department of Surgery, 4th Military Teaching Hospital, 5 Weigla Street, 50-981 Wroclaw, Poland; 3 Department of General and Vascular Surgery, Research and Development Centre, Voivodship Specialist Hospital in Wroclaw, Wroclaw, Poland; 4 Department of Otolaryngology, Head and Neck Surgery, Wroclaw Medical University, 213 Borowska Street, 50-556 Wroclaw, Poland

**Keywords:** Stroke, Endarterectomy, Quality of life, Stroke outcome, Carotid artery stenosis, Older patients

## Abstract

Strokes are one of the leading causes of death, morbidity, and disability worldwide, mainly among elderly people. It is also the third most common cause of years of life being lost, indicating a high risk of premature mortality. Revascularisation with endarterectomy (CEA) is effective in reducing the risk of death and strokes in patients with carotid artery stenosis, but the effect of invasive treatment on quality of life (QoL) still needs attention. To shed more light on the patients’ perspective on this health condition, we carried out a review of the literature which aimed to analyze the level of health-related QoL among stroke survivors, with special attention to patients who had been treated with CEA. Strokes significantly reduce the level of QoL, which may subsequently be improved in the course of treatment with CEA. Patients experience a reduced level of QoL in the early postoperative period, but at 1 year following CEA, the level of QoL remains stable and is similar to that of chronically ill patients. The domains of QoL which are most affected are physical and emotional functioning, which also serve as markers for decreased QoL in the long term. Older age and comorbidities are predictors of worse QoL. Stroke survivors require proper care both immediately after a stroke happens and during the long-term rehabilitation. Measurement of QoL and of the determining factors that contribute to a reduced level of QoL, as well as focusing on determinants of QoL in stroke survivors may help to reduce patients’ disability and improve their daily functioning in society as well as reducing the cost of health care.

## Introduction

Strokes are one of the leading causes of death, morbidity, and disability worldwide, mainly among elderly people [1]. Although mortality rates have decreased over recent decades, strokes remain the third commonest cause of mortality in more developed countries [2]. In 2012, strokes were also the third most common cause of years of life lost, indicating a high risk of premature mortality [3].

The World Health Organization (WHO) defines strokes as “rapidly developing clinical signs of focal (or global) disturbance of cerebral function, with symptoms lasting 24 h or longer or leading to death, with no apparent cause other than of vascular origin” [4]. Nearly, 80% of stroke cases are ischemic. Atherosclerosis is responsible for about 90% of cases of carotid artery stenosis. Ischemic strokes occur when the arteries become narrowed or blocked by an atherosclerotic plaque or an embolus at the level of carotid bifurcation.

Patients who have a stroke often suffer from many coexisting diseases. Gallacher et al. reported that nearly, 95% of stroke patients had one or more additional morbidity, which was almost twice the number of that in the control group. The most frequent coexisting diagnoses were hypertension, coronary heart disease, pain, and depression [5]. Cardiovascular diseases may further impair the condition of the affected vessels, leading to a gradual decrease in their lumen diameter. In addition, comorbidities lead to a decline in overall quality of life in this group of patients [6, 7].

Rapid treatment of a stroke is essential for survival. Patients with carotid artery stenosis are offered medical therapy, carotid endarterectomy (CEA), and endovascular treatment with angioplasty or stenting. Currently, CEA is the most common surgical procedure used to prevent future strokes from occurring [8, 9]. The beneficial effect of CEA on mortality and morbidity rates and the cost of treatment have been well examined, but the effect of treatment on quality of life (QoL) still needs attention. Stroke survivors require proper care both immediately after suffering a stroke and during the long-term rehabilitation. Measurement of QoL in stroke survivors and focusing on the determinants of QoL may help to reduce patients’ disability and improve their daily functioning in society. To shed more light on the patients’ perspective on the health condition, we carried out a review of the literature which aimed to analyze the level of health-related QoL among stroke survivors, with special attention to patients who had been treated with CEA. We performed a search of PubMed and Medline databases for studies including patients who had suffered a stroke, with special attention to assessment of QoL in stroke survivors and the efficacy of CEA. The search terms included the following: quality of life after endarterectomy, carotid stenosis, carotid angioplasty, and quality of life after stroke. The search was carried out from June to August 2016. In addition, electronic searches were supplemented by searching hard copies of the bibliographies of relevant articles. First, titles and abstracts were screened by two authors and next, and papers of interest were retrieved for full-text assessment.

## Who receives CEA?

Symptomatic patients with carotid stenosis experience neurological symptoms such as transient episodes of neurological dysfunction caused by focal brain, spinal cord, or retinal ischemia without acute infarction or stroke presented with sudden weakness of arms or legs, speech difficulty, severe headache, confusion, dizziness, or difficulty with balance or coordination. Symptoms worsen as the disease progresses and a stroke may even lead to sudden death [4, 10]. The burden of the disease and its severity reduces QoL, which may be further modified by the treatment options which are offered to stroke patients [11].

The method of stroke treatment is chosen based on the greatest benefits for an individual patient. The North American Symptomatic Carotid Endarterectomy Trial (NASCET) showed that CEA reduced the risk of a disabling stroke or death for symptomatic patients with stenosis over 50% [8]. Asymptomatic patients with carotid stenosis benefit less than symptomatic patients. The aggregate risk reduction of 53% was achieved for patients with asymptomatic carotid artery stenosis from 60% undergoing CEA when compared with patients who were offered aggressive medical treatment [12]. In addition, patients with severe heart disease, severe pulmonary disease, renal insufficiency, prior transient ischemic attack (TIA) or stroke, anatomical limitation, restenosis, and/or prior surgical treatment of carotid stenosis are at an increased risk of complications [9, 10].

## Tools for QoL measurement in neurologic patients

Measurement of QoL helps discover patients’ needs and offers them holistic treatment. Knowledge about QoL should complement other clinical findings. Self-assessed QoL is based on a subjective scale of severity of symptoms, so it brings additional information about efficacy and safety of treatment and, therefore, becomes one of the basic aims of the therapeutic approach. Assessment of QoL can contribute to the development of better and more efficient standards in the care and management of severe chronic diseases, including strokes. The level of QoL should be assessed at the onset of the disease, after the invasive treatment and periodically in the long-term, as a comparison of QoL can help to adjust treatment and restore independence.

There are several validated and standardised scales available for use among patients with carotid stenosis and stroke survivors [13]. The original 49-item scale and its shortened version—the 12-item Stroke-Specific Quality-of-Life Scale (SSQOL)—address both health-related QoL domains and stroke-specific domains including speech, mobility, vision, and upper extremity function [14]. The Nottingham Health Profile was designed to measure subjective health status. It is used to determine the effect of a given disease on a patient’s QoL, but it is not disease specific [15]. The 36-item short-form General Health Survey (SF-36) questionnaire includes one multi-item scale. It is used to evaluate limitations of health domains such as physical, social, role and emotional functioning as well as bodily pain, general mental vitality, and general health perceptions [16]. Cabral et al. found that SF-36 is more suitable for evaluating QoL in patients in the chronic phase after a stroke [17]. The sickness impact profile (SIP) is based on sickness-related behaviour. It is a 136-item scale consisting of 12 subscales. Due to its length, SIP is more suitable for cross-sectional assessment than for the assessment of individuals [13, 18]. In severely ill patients, functional status is often determined by the ability to care for oneself and the possibility of living independently. The Katz Activities of Daily Living scale evaluates basic personal activities of daily living, while the Lawton Instrumental Activities of Daily Living scale gives information about a person’s ability to live in the community [19, 20]. The EuroQol is another generic scale for the measurement of health-related QoL. It is simple and short as it measures only five health dimensions (mobility, self-care, social functioning, pain, and mental functioning). The EuroQol evaluates similar domains of health to the SF-36 questionnaire. The QoL results measured by these scales correlate closely, except in the category of mental functioning [21].

The questionnaires given above are only examples of the available diagnostic possibilities for the assessment of QoL in stroke patients. Researchers are still working on new and more accurate scales or adapting existing scales for use among stroke patients, including the assessment of the impact of invasive treatment such as CEA. It should be kept in mind that the choice of a scale for QoL measurement should be based on the unique diagnostic needs of the subjects being studied, their clinical characteristics, the research questions, and the feasibility of the scale.

## QoL drops in patients with carotid stenosis and after a stroke

Large studies show that patients suffering from carotid stenosis experience reduced QoL. The Oxford Vascular Study shows that TIA patients had significantly lower QoL than controls and that the level of QoL was stable during the 5-year follow-up [22]. Vlajinac et al. reported significantly lower SF-36 scores in the scope of physical, social, emotional, and mental functioning in patients with symptomatic carotid disease than those in the population in general. They also found that the progression of the disease reduced physical functioning and did not affect other domains of QoL [11]. In the study performed by Dardik et al., a group of patients with carotid artery stenosis ≥65% was compared with normal and chronically ill groups before CEA and after the procedure using the SF-36 questionnaire. Only role limitations due to physical problems was significantly worse in patients before CEA than in the normal group, while mental health, bodily pain, and general health perception was better than in the group of people with chronic diseases [23].

The level of QoL changes after a stroke. It decreases straight after an ischemic event and then improves regardless of the method of treatment, but remains lower than in the healthy population; however, the long-term QoL depends on many factors. In the Oxford Vascular Study, the EuroQol score improved significantly between the measurement at 1 month and at 6 months after the stroke. The lowest QoL level was reported by severe stroke patients and the highest by minor stroke patients. In stroke patients, the average EuroQol score was significantly lower than in controls at 1 month and remained significantly lower at 5-year post-event [22]. In the study by Prlic et al. carried out using the SF-36 questionnaire, the poorest results of QoL were also obtained 30 days following the stroke and recovery was achieved in 6 months; however, the stroke had a significant effect on the QoL of the affected subjects [24]. The KOSCO study performed at 6 months after a first-ever stroke showed that years of education, motor, ambulatory function, and language function are positive predictors for QoL in patients with an ischemic stroke [7]. Grabowska-Fudala et al. observed stroke patients who received only thrombolytic treatment for a period of 1 year. The median SSQoL score and the number of patients reporting good health-related QoL increased insignificantly when measured at 3 and 12 months following discharge. The level of QoL was associated with stroke severity and baseline functional disability [25]. Lopez-Bastida et al. found that post-stoke patients have a significantly lower QoL at 1, 2, and 3-year post-event as measured by the EuroQol score when compared with the general population [26]. Similarly, Katona et al. revealed that the health-related QoL in stroke patients living at home remained at the same low level for 2.5 years following discharge while support such as in-patient neurological rehabilitation decreasing functional deficits resulted in an improvement in emotional functioning in this group of patients [6]. Prlic et al. reported that patients who stayed with their families after a stroke had better physical and mental health even than those before a stroke, emphasising the role of support in this group of patients [24].

Post-stroke depression is one of the long-term adverse psychosocial consequences in stroke survivors. It develops in about 30% of patients during the first year following a stoke. Carod-Artal et al. found that post-stroke depression more often affects women than men. Depression was also associated with cognitive functioning and dependence in the instrumental activities of daily living, which altogether contribute to poor QoL [27].

The presence of lobar cerebral microbleeds during the acute phase of an ischemic stroke further damaged physical, social, and emotional functioning [28]. Osipenko and Marochkov observed that symptoms affecting QoL depend on the type of pathological changes within carotid arteries. Patients with stenosis suffered predominantly from pain, discomfort, and motility, while patients with pathological kinking suffered from pain, discomfort, anxiety, and depression [29].

The difference between men and women in perceived QoL following a stroke is not clear. In men, physical functioning, vitality, and mental health were worse than in the general population, whereas women reported a lower level of QoL in physical functioning, role, general physical health, vitality, and mental health than in the general population [30]. There are also reports showing that the level of physical and mental functioning as measured by the SF-36 was better in men than in women [24]. Women also showed a trend towards an improved change in health assessed as measured with the SP-36 after CEA [23].

## Endarterectomy improves QoL

CEA is one of the treatment options for patients suffering from an inadequate blood supply to the brain though the internal carotid artery. During the procedure, the atheromatous plaque of fatty deposits that narrow the lining of the artery is surgically removed. There are several techniques used during surgery. Conventional CEA is done after exposing the carotid artery by a longitudinal incision on the side of the patient’s neck, followed by eversion or longitudinal endarterectomy. In the case of stenosis, closure of the artery may be done with a patch to expand the lumen diameter. Alternatively to CEA, carotid artery stenting may be performed [10].

Invasive treatment is recommended in symptomatic patients with carotid artery stenosis exceeding 70%. It may also be performed in symptomatic cases with stenosis over 50% and in selected subgroups of patients with asymptomatic stenosis. It is also recommended that the procedure should be carried out not later than 2 weeks after the onset of a stroke. It is well documented that CEA reduces the risk of disabling strokes or death for patients with severe carotid artery stenosis [8, 9, 12]. Postoperative assessment of QoL is one of the recognized measures of the results of surgical treatment. Knowledge about QoL in patients after revascularisation is still insufficient, despite intensive research which has been conducted recently on this topic. To date, only two randomized trials have been conducted. The results of many other studies are difficult to compare due to differences in methodology [31].

Abelha et al. studied a group of 63 patients with carotid artery stenosis of ≥65%. The Katz and Lawton scales showed a significant increase in dependence in activities of daily living 6 month following discharge when compared with the results recorded within the first 24 h after admission to the hospital. The SF-36 scale showed that 63% of the study cohort reported an increase in QoL, while 11% of them reported a decrease in QoL. The SF-36 scores reported by stroke patients were worse than those of the healthy urban population except for bodily pain, but similar to those of non-cardiac surgical patients [32].

Dardic et al. evaluated QoL in 50 patients with symptomatic carotid artery disease before undergoing CEA and at 3 months following surgery. They revealed that the SF-36 overall “change in health” score improved significantly after CEA when compared with the preoperative score, but changes in all QoL domains were insignificant. The levels of physical functioning and role limitation–physical were similar to those of the chronically ill group, while the level of social functioning, mental health, bodily pain, and health perception was similar to the scores in the healthy group [23]. In addition, asymptomatic patients with stenosis greater than 75% experienced an improvement in cognitive function after CEA. Cognitive function increased significantly between the preoperative measurement and at 3 months following CEA in patients with and without dementia [33].

The prospective assessment of QoL in patients presenting neurological symptoms of the contralateral internal carotid artery at 1 week before and 3 months after CEA was conducted by Vriens’ team. The patients in the study were classified into three groups according to their preoperative neurological dysfunction. The first group included subjects with no cerebral symptoms, having only ocular symptoms or being asymptomatic. The second groups consisted of patients with transient symptoms of TIA. The third group included patients with permanent cerebral symptoms and a major stroke. For the assessment of health-related QoL, the SIP questionnaire was used. At baseline, the SIP scores were significantly higher (indicating poorer functional health) in the stroke group than in patients from the two other groups. Three months after surgery, the level of QoL of the total study group had not changed. An improvement was observed in patients with an occlusion of the contralateral internal carotid artery and in those who needed an endovascular shunt during CEA [34]. The association between the severity of damage to the brain and the degree of improvement of QoL was also confirmed by Svedonov’s team. A comparison of health-related QoL between the preoperative and postoperative measurements revealed that asymptomatic and minimally symptomatic patients showed greater improvement (in six out eight scales) than those with a major stroke (in one of the eight scales) [35].

A comparison between patients suffering from a minor cerebral ischemia who underwent either CEA or were treated with the best medical therapy revealed no significant differences in the eight SF-36 scales except overall health, which was better in the CEA group. A higher percentage of patients treated with CEA were convinced that their treatment was successful and their health had been improved by the treatment when compared with patients treated non-invasively [36]. The results of many other studies show the superiority of CEA over conservative treatments in terms of efficacy and improvement in QoL [37, 38].

Most of the studies confirmed that QoL becomes stable at a year after CEA, but does not return to the values characteristic for the general population [30, 37, 39]. Improvement of QoL after CEA was maintained even after 8–11 years following surgery [40].

## Predictors of QoL in stroke patients treated with CEA

An ischemic stroke is a serious condition which may lead to permanent damage to the brain tissue. Revascularisation restores the blood flow to the affected areas and contributes to preventing the occurrence of a stroke in the future, although some symptoms of the stroke may persist. The studies conducted among stroke patients show that long-term health-related QoL depends on many factors, including the severity of the stroke, the patient’s health condition, treatment, and rehabilitation as well as socio-economic factors.

Older age is a widely discussed risk factor for poor QoL as it is associated with a greater number of coexisting diseases, decreased mobility, and the progression of atherosclerosis among others. Middleton et al. observed that in stroke patients undergoing CEA, an older age correlates with worse physical functioning [30]. On the contrary, Abelha et al. did not find any association between decline in QoL and patient characteristic [32].

Severity of stroke is a recognized factor that translates into a reduced level of health-related QoL [34, 35]. Moreover, as revealed by Vriens et al., significantly more intraoperative shunting during CEA is used in the group with a permanent neurological deficit and this is associated with significantly higher preoperative SIP scores (poorer functional health) in this group of patients than in patients who did not need intraoperative shunting [34]. Shunt insertion was found to be associated with cognitive decline on the first day after CEA when compared with assessment before surgery [27]. Furthermore, Lloyd et al. found that the occurrence of microembolisations increases the risk for the decline in cognitive function [41].

Events that complicate the perioperative and postoperative periods may affect QoL, as the most serious complications may lead to significant long-term disability. Cranial nerves injury is the most common neurologic complication of CEA. The incidence of cranial nerves injury varies widely, from 3 to 30% of cases undergoing CEA. A large percentage of these injuries resolve themselves spontaneously within a few weeks; however, neurologic symptoms may persist in 7–12% of patients, depending on the degree of damage [42]. Symptoms of cranial nerve injury depend on the damaged nerve and in severe cases may result in the use of a feeding tube and/or a tracheostomy. Despite the severity of this complication, Hye et al. did not find any significant differences in SF-36 scores between patients who developed cranial nerve injury and those who did not at 2 and 4 weeks and 12 months after CEA. Although patients with cranial nerve injury demonstrated greater difficulty with eating/swallowing at 2 and 4 weeks following CEA when compared with uncomplicated cases, at 1 year, no significant differences in any parameter were observed [39]. Other researchers also confirmed the lack of association between cranial nerve injury and the level of QoL in patients subjected to CEA [43].

Taking into account other complications of the CEA procedure, Dardik et al. found that a positive change in health assessed with SP-36 was significant only in patients who did not have any postoperative complications, whereas patients with a complicated postoperative course reported decreased emotional functioning. Preoperative contralateral carotid artery occlusion decreased the health perception score, while a preoperative stroke had no impact on QoL [23]. Other researchers found no impact of the duration of the procedure and the type of anesthesia on QoL outcome [29, 44]. QoL is also reduced in patients who require reoperation [40].

## Does QoL depend on the surgical technique used for carotid artery revascularization?

Although currently, CEA is the gold standard for carotid artery revascularisation performed with the aim of preventing strokes, other surgical techniques draw the attention of surgeons, because many patients are considered poor candidates for CEA. In addition, there are some modifications in performing CEA as well that can be used in the case of anatomical difficulties or large plaques [45–48]

Carotid artery stenting (CAS) was developed as an alternative to CEA for patients at a high risk of adverse outcomes from surgical CEA. The SAPPHIRE trial showed that CAS with an embolic protection device gave comparable outcomes in terms of prevention of death, stroke, or myocardial infarction among high-risk patients with risk factors such as those who were older, or who had comorbidities or anatomical difficulties [46, 49]. Major adverse events after CAS are well within an acceptable range (4.4%) [50]. More cranial nerve palsies appear after CEA than after endovascular treatment [46]. Despite promising results, endovascular treatment still requires long-term evidence obtained in adequately powered trials to demonstrate its benefits also in terms of QoL. It should also be mentioned that CEA has been used for decades, whereas experience and knowledge about CAS outcomes are smaller.

The first comparison of health-related QoL following CAS and CEA was performed in the framework of the CaRESS trial. A single assessment of QoL changes was performed at 1 year following treatment. In the CAS arm, a greater decline in QoL was observed than in the CEA arm, but there were no significant differences between CAS and CEA patients [51]. Randomized assessment of QoL was conducted in the framework of the SAPPHIRE Trial. QoL was assessed at baseline, at 2 weeks, and during additional follow-up visits at 1, 6, and 12 months. Patients from the CAS group reported better scores at 2 weeks for the SF-36 role physical scale than patients from the CEA group. No other significant differences were found for other scales between study groups and at any other time points. When taking into account the results of the disease-specific modified Likert scales, the only differences between CAS and CEA groups were observed at 2 weeks. Patients from the CAS group reported less difficulty eating or swallowing, less difficulty driving, and less neck pain, but at 1 month, no differences between the study groups were observed [52]. The CREST study had a similar design to the SAPPHIRE Trial and the results were consistent with the latter. Health-related QoL was better in CAS patients than in CEA patients only during the early recovery period. No differences were observed at 1 year [43]. Better QoL in CAS patients than in CEA patients shortly after surgery can be explained by the less invasive nature of CAS as compared to CEA.

## Conclusions

Strokes negatively affect QoL, but its level can be restored by proper treatment. CEA improves outcomes and reduces the risk of a stroke in patients with carotid artery stenosis. At 1 year after CEA, the level of QoL becomes similar to that of chronically ill patients. The physical and emotional domains are the most affected components of QoL. Reducing disability before discharge should be a priority as physical functioning has a major effect on health-related QoL in stroke patients.
